# Exploring ecologies of resilience in Filipino frontliners’ experiences of stress and strength

**DOI:** 10.3389/fpsyg.2026.1814261

**Published:** 2026-06-29

**Authors:** Angelique Pearl Virtue Villasanta, Junix Jerald Delos Santos, Liane Peña Alampay, Christelle Johanna Reganion, Clarisse Aeaea Kilboy, Anna Czarina Mikael Isaguirre, England Danne Castro, Teresita Panganiban, Karina Therese Fernandez, Mary Janneke Agustin, Iris Emilie Trinidad

**Affiliations:** 1 Department of Psychology, Ateneo de Manila University, Quezon, Philippines; 2 Department of Psychology, School of Teacher Education and Liberal Arts, University of Baguio, Baguio, Philippines; 3 De La Salle University, Manila, Philippines; 4 FriendlyCare Foundation Inc., Mandaluyong, Philippines

**Keywords:** collective resilience, frontline worker, global south, resilience, stress

## Abstract

This study responds to two conceptual and empirical gaps in resilience literature. Firstly, dominant resilience literature has viewed resilience as an individual psychological capacity or internal resource. Secondly, there is a dearth of qualitative studies that particularly examine the lived experiences and meanings of resilience for frontline workers (FLWs) from the Global South, especially in the Philippines. As a contribution, this study explores Filipino FLWs experience and meaning-making of resilience as a product of dynamic interactions of the individual with broader collective, social, and organizational systems. More specifically, this study explores different ecologies that shape FLWs’ resilience: their individual ways of coping, relationships with peers and colleagues, workplace systems, family and community support, cultural values, and spirituality. Through reflexive thematic analysis following a collaborative qualitative data analysis framework, we identified themes regarding the FLWs’ experience of being a frontline worker, their narratives of stress, and the meanings attributed to resilience. Results describe three salient narratives of stress: stress as a normal part of everyday life, as being “for the weak,” and as a forbidden cognitive mindset that should not be entertained. Salient meanings of resilience include collective strength and strength from God. These findings expand the understanding of resilience to include relationality, embodiment, cultural values and spirituality, which provide implications for programs, interventions, and how institutions can be of support.

## Introduction

Resilience is widely defined as an individual’s capacity to adapt positively in the face of adversity, stress, or trauma. The American Psychological Association (2018) describes it as the process and result of effectively coping with and adapting to challenging life situations by demonstrating mental, emotional, and behavioral flexibility in response to both external pressures and internal demands. Other definitions of resilience recognize the role played by culture, values, environment, and context. Since the first “wave” of resilience studies in the 1970s, understanding resilience has evolved from the identification of protective and risk factors, to the use of dynamic systems perspectives to explore the role of context, cultural values and practices, and collectives in shaping resilience (Masten, 2012, 2014). For instance, existing literature has pushed for the recognition of cultural, social, and institutional contexts as valuable determinants of mental health and resilience (Naeim and Basharpoor, 2026). In the work of Ungar (2011), resilience is explored not so much as an individual trait or capacity, but as a quality of people’s social and physical ecology.

This study adapts an ecological and systems perspective in the understanding of resilience to explore the meanings and experiences of resilience among Filipino frontline workers (FLWs) who respond to crises and emergencies in the areas of disaster response, healthcare, social work and early childhood care and development (ECCD), firefighting, and law enforcement. In doing so, this study aims to contribute toward the empirical gap of understanding meanings of resilience coming from frontliners in a Global South and disaster-prone country. As a conceptual contribution, this study explores resilience as beyond individual traits and capacities, by looking at FLWs personal, social, community, and cultural ecologies: their individual ways of coping, their ways of relating with peers and colleagues, the presence of family and community support, shared cultural values, and spirituality.

Recently, resilience has become an increasingly critical area of study, particularly in the context of global crises (Denckla et al., 2020; Esmail et al., 2021; Popham et al., 2021). In the face of crises (such as the COVID-19 pandemic), frontline workers (FLWs) play a vital role in delivering essential services (Musheno et al., 2021). FLWs stand at the forefront of these challenges as they navigate heightened exposure to stressors such as burnout, trauma, and systemic constraints; all while responding to crises and emergencies (Alqahtani et al., 2024; Di Giuseppe et al., 2021). In these high-risk occupations where exposure to adverse events or potentially traumatic situations is likely, previous literature has highlighted the importance of exploring both individual and collective experiences of resilience (Brassington and Lomas, 2020), along with relational, social, and cultural systems that shape it. This perspective is aligned with recent developments in the field of resilience studies (Masten, 2021), where resilience is understood as a dynamic capacity shaped by constant interactions between people’s internal capacities, their social relationships, and their involvement with broader systems (e.g., family, community, workplace, etc.).

### Global and regional understandings of resilience among FLWs

Globally, studies on resilience among FLWs emphasize the interplay between individual psychological factors (e.g., coping mechanisms, emotional regulation) and external support systems (e.g., organizational policies, peer networks; Bartuska and Marques, 2021; Dhillon and Das, 2024). This suggests that resilience is a dynamic process influenced by various factors (i.e., environmental, relational, and personal). For instance, research in collectivist cultures highlights the significance of relational and communal dimensions in fostering resilience, where community support, shared values, and collective coping strategies play pivotal roles (Kuo, 2013; Murray and Zautra, 2012; Ungar, 2015).

Resilience in the Global South is often understood through a collectivist and ecological lens, where relational support systems buffer adversity (Hechanova et al., 2015, 2022, 2023; Rockenbauch and Sakdapolrak, 2017; Wright and Masten, 2015). This perspective complicates Western models that emphasize individual agency. For instance, resilience in Asian contexts is viewed as interdependent, and sustained by interconnectedness, religious faith, and community cohesion (Chua et al., 2019; Fernando, 2012; Suleeman, 2021; Thomas et al., 2016). Specifically among FLWs in Asia, cultural norms reinforce collective resilience and support communities’ recovery from crises. This emphasizes the importance of community empowerment, collective efficacy, and co-constructed support systems in the face of large-scale challenges (Atallah et al., 2021; Brodsky and Cattaneo, 2013; Garabiles et al., 2022).

### FLWs in the Philippine context

Frontline workers in local government units in the Philippines perform essential services that entail direct interaction with people and communities, in order to provide crucial support in day-to-day situations and emergency or crises events. Frontline work spans different units and agencies to cover important community needs, such as healthcare, emergency services, safety and security, provision of social services, and child support and protection. In the Philippines, FLWs include police workers, firefighters, disaster and emergency response personnel, health care workers, social workers, and early childhood care and development workers. While these groups may fall under different agencies, they closely collaborate in addressing community needs and emergencies. For instance, collaborations among the disaster and emergency response personnel, health care workers, and social workers have been crucial during the COVID-19 pandemic, in responding to COVID-19 patients and in rolling out vaccination. In other instances, early childhood care and development workers have been deployed alongside emergency responders to further augment the support that can be provided to the community. While each frontline unit may have unique experiences, different exposures to stress, carry particular roles and responsibilities, or have specific institutional contexts that can be explored in future studies, this study presents common experiences across the different FLWs to address an important empirical gap in this area.

In the Philippines, FLWs face unique sociocultural and systemic challenges that further complicate the ability to be resilient (Haldane et al., 2022). Resource limitations, frequent exposure to natural disasters, and cultural obligations (e.g., familial expectations) create a multifaceted backdrop for Filipino FLWs.

Despite these difficulties, resilience emerges not only as a necessity but also as a cultural strength deeply rooted in Filipino values (Bistis-Nadala, 2024; Isidro and Calleja, 2021). Concepts such as *bayanihan* (communal unity), *pakikipagkapwa* (shared humanity), and *malasakit* (compassion) exemplify how resilience is experienced as relational and collective, and is embedded in the Filipino way of life (Isidro and Calleja, 2021). These cultural constructs support the interconnectedness of individual and collective resilience, with personal coping strategies often reinforced by communal support systems (Adviento and De Guzman, 2010). Filipino psychologists emphasize *kapwa* (shared inner self) as a uniquely Filipino cultural value that underpins resilience (Aguiling-Dalisay, 2013; De Guia, 2013). From this perspective, resilience is not only about self-reliance but also about mutual support and shared burdens within families and communities. Resilience is also tied to spirituality and meaning-making (Jocson and Garcia, 2017; Lee-Chua, 2022; Narimani and Naeim, 2025; Yeung and Martin, 2013). It involves drawing strength from faith, finding purpose in adversity, and leaning on religious or spiritual practices to cope with challenges.

### Ecological systems perspective to understand meanings of resilience among FLWs

In this current paper, we adopt an ecological systems perspective to explore the interplay between individual and collective resilience among Filipino FLWs that emphasizes the cultural and contextual dimensions that shape resilience (Bronfenbrenner and Morris, 2006; Ungar, 2011). While extant frameworks on collective resilience have contributed to the literature by focusing on relational dynamics and resource mobilization, these often prioritize structural and operational processes (e.g., social networks and institutional supports) over deeply embedded cultural and spiritual factors (Kulig et al., 2013; Norris et al., 2008).

In the context of Filipino FLWs, this current study seeks to explore the role of individual and shared narratives of resilience. In addition, by drawing insights from studies on workplaces and organizations (i.e., the impact of organizational culture and relational dynamics on resilience), we aim to bridge the gap between macro-level conceptualizations of resilience and micro-level workplace dynamics (Avey et al., 2009; Kuntz et al., 2017). This extends the current literature by integrating cultural and spiritual dimensions into frameworks for organizations and communities. Although studies have examined how philosophical and spiritual worldviews contribute to professional resilience, this connection remains underexplored in the literature (Rush et al., 2023). This study thus offers a richer and more contextually grounded perspective on resilience in resource-constrained and culturally diverse settings. By centering the voices of Filipino FLWs, this research seeks to contribute to culturally grounded and nuanced understandings of resilience and inform interventions that put the lived realities of FLWs front and center. Therefore, we ask:

1. How do Filipino FLWs experience individual and collective resilience?
2. What are the meanings of individual and collective resilience for Filipino FLWs?

## Method

### Research design

This study utilized a qualitative research design. Qualitative data was gathered through (a) key informant interviews, (b) walking interviews, and (c) focus group discussions (FGDs). This study is part of a larger government-funded research project that aims to develop and evaluate a resilience program for Filipino FLWs. The research procedures were reviewed and granted ethics clearance by Ateneo de Manila University.

### Setting

The Filipino FLWs in this study were employed by the local government of a highly urbanized city, with one of the largest populations in the National Capital Region of the Philippines (Philippines Statistics Authority, 2021). Given its proximity to rivers and waterways, the city’s drainage system is problematic and flood-prone. In recent years, the city was declared under a state of calamity during several major typhoons or storms (e.g., in 2024, during Super Typhoon Carina [international name: Gaemi]; in 2013, during Severe Tropical Storm Maring [international name: Kompasu]. In 2009, during Tropical Storm Ondoy (international name: Ketsana), almost all of its barangays were severely flooded with waters reaching as high as 16 to 20 feet (Soriano, 2009). The city has also experienced one of the most devastating fires in the history of the Philippines. Despite these, the city has received distinctions and awards for good local governance and in relation to issues such as violence, safety, and health response systems.

### Participants

Eighty-four (84) FLWs across six frontline units—i.e. disaster response, health care, police, early childhood care and development (ECCD), social work, and fire—participated in key informant interviews, focus group discussions, and walking interviews (see Table 1 for breakdown of participant per frontline unit). As one of the larger frontline units, ECCD augments the social work unit in times of disasters and emergencies. Representatives from each frontline unit were briefed regarding the criteria for participation. They identified prospective participants from their own unit. These participants were contacted by the research manager for screening.

**Table 1:** *N* of participants in the key informant interviews, focus group discussions, and walking interviews across six frontline units.

Frontline unit	Disaster response	Health	Police	Early childhood educators	Social work	Fire	*n* per data collection type
KIIs	1	1	1	1*	1	1	5
FGDs	10	14	16	11	9	12	72
WIs	1	1	1	1	1	2	7
*n* per frontline unit	12	16	18	13*	11*	15	

*The key informant participant for the early childhood education unit is the same as the key informant for the social work unit.

Participants met the following inclusion criteria: (a) age is between 18 to 65 years old, and (b) worked for at least 1 year in their respective frontline unit. For key informant interviews, participants met the following additional inclusion criteria: (a) are unit heads of their respective frontline unit, (b) possess deep knowledge and institutional memory about their frontline unit, (c) have a depth of familiarity with the nature of work of the FLWs in their unit, and the stresses and challenges faced by the FLWs. Prospective participants were excluded from the study if they were screened to have low mood or elevated anxiety in the past two weeks as indicated by the Patient Health Questionnaire-2 (PHQ-2; Kroenke et al., 2003) and the Generalized Anxiety Disorder-2 (GAD-2; Kroenke et al., 2007). Informed consent was sought through an online form, prior to inclusion in the interviews or FGDs.

The majority (58%) of participants in the study were women. Almost half of the participants were either in the age group of 31–35 (20.4%) or 41–45 (20.4%). Very few participants were aged 56 or older (2.2%). The years of service of the participants ranged from 3 years to 27 years.

### Data collection procedures

#### Key informant interviews (KIIs)

Five KIIs were conducted. A KII was conducted for each of the frontline units, except for ECCD and social work which shared the same key informant. The KII explored the role and responsibilities of the participant as a unit head, the key events and challenges encountered by their frontline unit, the nature of the frontline work done by the unit, and the stresses and ways of coping of the FLWs. The KIIs lasted between 1 h and 30 min to 2 h and 30 min.

#### Focus group discussions (FGDs)

Nine FGDs were conducted across six frontline units. Two FGDs each were conducted for the units of healthcare workers, police, and firefighters, while ECCD, social work, and disaster response had one FGD each. The FGDs centered on FLWs’ experiences of day-to-day work, the challenges they encounter in their work, and the impacts of these challenges on themselves and their relationships with workmates, loved ones, and family members. The FGDs also explored challenges they encounter outside their role as an FLW (e.g., challenges at home, or challenges in other contexts). It also explored their ways of coping, and the support they need from the local city government, their immediate supervisors, colleagues, and family. The FGDs lasted between 1 h and 20 min to 2 h and 40 min.

#### Walking interviews (WIs)

Seven WIs were conducted across the six units, with two WIs for the firefighters. Similar to the KII and FGD, the WIs explored the nature of work, roles, responsibilities, and challenges encountered by FLWs in their typical day-to-day. Unique to the WI is the walking component, where participants led the researcher through the typical routes and places they go to as part of their frontline work. As they move through the route, the interview probed into the usual events that would happen in each space, the tasks or activities done by the participants in those spaces, the individuals they encounter there, and the usual emotions that come up in those spaces. After the walking component, the WIs are synthesized by asking the participant to identify spaces of relaxation or wellness, and spaces of stress or distress. The WIs lasted between 1 h to 1 h and 40 min.

The KIIs, FGDs, and WIs were audio recorded and transcribed verbatim. Observers’ notes were also collected and transcribed. Identifying information (e.g., names, designations) was removed during transcription to ensure confidentiality.

### Data analysis

Data were analyzed using reflexive thematic analysis (Braun and Clarke, 2019), following a collaborative qualitative data analysis framework (Richards and Hemphill, 2018). Reflexive thematic analysis served as a guide into deep and reflective engagement with the gathered data, while the collaborative qualitative data analysis framework organized how researchers worked together through analyzing a large data set in a manner that valued rigor and trustworthiness in the produced analysis. Given that this study employed a combination of two different methods, we outline the phases of the analysis to “own” the orientation, assumptions, and data analysis decisions made by the researchers (Braun and Clarke, 2023).

#### Preliminary organization and planning

Prior to data analysis, an orientation was conducted with all researchers regarding data collection, tackling the values and assumptions that underlie the research. After data collection, a second orientation was conducted to review the constructivist orientation and systems perspective employed in the study and revisit the research questions. During this phase, researchers agreed on the focus on the experience and meanings surrounding stress and resilience, their ways of coping with encountered adversity—along with resources that they find supportive of this, and any barriers or challenges to their ability to cope or manage. Given the systems perspective employed in this research, the team noted the importance of looking at the individual and the different contexts they navigate (e.g., family, work, community, and environment).

#### Open coding and creation of the coding guide

This phase involved researchers familiarizing themselves with the transcripts through repeated active reading and memo writing. Four researchers were assigned to analyze the transcripts of interviews and FGDs that they conducted, while two researchers read through and analyzed all transcripts. In this phase, the research team met to discuss initial impressions of the data and to share how each is working through the initial coding process. The researchers coded for both semantic and latent meanings.

The researchers’ personal memo notes and codes, and the discussions from the team meetings were used to develop the coding guide. The intention of the coding guide was to reasonably manage the step of coding a large data set in a systematic and thorough manner. This step produced two revisions to the coding guide, reflecting the iterative nature of this phase.

#### Coding and collaborative identification of themes

In this phase, researchers analyzed how the codes cohere and how the analysis might respond to the research questions posed by the study. This phase ended with the generation of preliminary themes.

#### Consultation and review of themes

The preliminary themes, theme descriptions, and key data extracts were presented twice within the research team. After revisions, it was presented once to the larger project team for their feedback. It was presented three times to a smaller sample of participants and community partners for their further input and to validate whether the construction of the themes made sense. After this, the themes were finalized and written up. This current analysis presents themes that were shared across and validated by all frontline units involved.

## Results

Through the analysis, we identified themes about FLWs’ experiences of their work, along with their meanings of stress and resilience (see Table 2 for a summary).

**Table 2:** Summary of generated themes and sub-themes regarding FLWs’ experience and their meanings of stress and resilience.

Section	Themes and sub-themes
The experience of being a FLW	Frontline work as an overwhelming workload
	Frontline work as psychologically distressing
	Frontline work as unappreciated work
	• A thankless job
	• Lacking in benefits and support
	• Differential valuing from supervisors
	Frontline work as a sacrifice and a duty
	Frontline work as bitter sweet
FLWs’ narrative of stress	Stress as a normal part of everyday
	Stress as “for the weak”
	Stress as a forbidden cognitive mindset that should not be entertained
FLWs’ meanings of resilience	Resilience as ability to endure
	Resilience as ability to keep one’s cool
	Resilience as ability to regenerate
	Resilience as ability to separate from work
	Resilience as strength from god
	Resilience as collective strength

### The experience of being a FLW

#### Frontline work as an overwhelming workload

In their day-to-day, FLWs contend with a heavy workload characterized by long hours of responding to emergencies or dealing with requests, paperwork, and many other responsibilities, often extending beyond their regular work hours. FLWs describe how there is a sense of work as never-ending, and, in their words, “having no “time in” and “time out”.” Aside from the long hours, FLWs contend with physically and emotionally demanding tasks, such as when responding to fires, disasters, and medical emergencies, or dealing with irate clients and hearing emotionally heavy accounts of poverty, grief, or abuse.

For FLWs in the field, part of their day-to-day involves assessing, navigating, and dealing with potentially threatening situations or situations that pose a direct risk to their safety. Compounding this risk is a lack of resources and equipment, where FLWs are sometimes left to improvise (e.g., using garden gloves instead of standard gloves). Part of FLWs’ work in the field also entails working with and managing bystanders who interfere with operations. FLWs who are assigned to desk-based work contend with needing to serve multiple clients or work through several requests and paperwork. On top of these, both FLWs in the field and the office, also have to comply with government-required reports and show up in meetings or training seminars. This heavy workload eats into lunch breaks and sometimes even into restroom breaks, work leaves, and time with family.

#### Frontline work as psychologically distressing

FLWs talked about the emotional and cognitive toll that their work takes on them, and how it stays with them long after. Field FLWs talk about harrowing sights of corpses and terrible injuries that they encounter in their work. They share how some images, sounds, and even scents stay with them even months or years after. For FLWs in the office, they share about the burden of hearing account-after-account of grief, death, violence, and abuse, and how they feel heavy and burdened afterwards. FLWs also talk about feeling vigilant when they hear alarms from ambulances or fire trucks, even when they are at home and not “on duty.”

When facing emotional distress, FLWs feel wary of disclosing to their supervisors or the human resources unit because of fears that it might have repercussions on their employment or evaluation. For other FLWs, they perceive that psychological support is not available in the workplace.

#### Frontline work as unappreciated work

##### A thankless job

There are instances where it becomes apparent for FLWs that their jobs are undervalued by community members, or sometimes even directly criticized or disrespected. FLWs shared being perceived by the community as being untrained, unskilled, or incompetent in their work, or simply not doing enough. In some instances, bystanders have taken photos of FLWs in the field and have posted them online along with negative comments, drawing more flak from commenters.

##### Lacking in benefits and support

Financially, FLWs see their jobs as lacking in terms of providing them with financial security for themselves and their family. For most of the FLWs, getting into a tenured position is not even possible. FLWs also talked about how they would need to think about other sources of income beyond their job.

##### Differential valuing from higher-ups

FLWs also talked about the salient effects of their higher-ups’ evaluation of their skills and competence, in that FLWs who were seen as more competent received more workload. There are instances where they also take on the load of “less competent” colleagues, leading to resentment in the workplace. On a broader level, FLWs also identified instances where entire frontline units are seen as more competent compared to other units, which leads to more opportunities, support, and funding devoted to those units, while other units perceive that they remain underfunded or unappreciated.

#### Frontline work as a sacrifice and a duty

Understanding that their work is difficult yet important, FLWs make meaning of their work as both a sacrifice and a duty. They speak of their work as a commitment, a service, a purpose, and a calling. FLWs recognize how their work entails making several sacrifices—such as sacrificing time with family (e.g., missing out on important family events, family vacations, and spending time with their partner and/or children), and letting go of more high-paying job opportunities. More deeply, FLWs see their work as a sacrifice of themselves—sacrificing their lives for the good of the community and the country. Ultimately, these sacrifices are seen as intertwined with seeing their work as a duty, or, in their words, an “oath” that they took and carry with them even when they are not on duty or not wearing their uniform. For them, being a FLW is their life, and it does not end when they clock out.

#### Frontline work as bittersweet

Frontline work is an intertwining of emotional dualities. Salient in the accounts of the FLWs is the intertwining of opposite affects—happiness and sorrow; pride and degradation; loneliness and community; and hope and despair—often coded in the same spaces of work. For instance, FLWs have shared about the space of the ambulance as happiness and sorrow intertwined—with memories of driving all around the country and visiting scenic sights along the way, but also a space where they encountered the direst of emergencies and lost lives. FLWs also spoke about the deep pride that they feel when people thank them for their service, but also the degradation they feel from being looked down upon. During the COVID-19 pandemic, FLWs talked about how the isolation tent was a witness to their moments of despair at its height, and how the same tent was there in their moments of hope during the rollout of vaccinations.

### FLWs’ narratives of stress

#### Stress as a normal part of everyday

FLWs speak of stress as part of their everyday. For FLWs, stress is an inherent aspect of the job that they implicitly accepted when they took on the position. For them, stress is a given part of their work conditions, knowing about the long hours, responsibilities, and the nature of their work. Stress is also seen as part of dealing with the demands of the higher-ups, community, and colleagues. Latent in this narrative of stress is its justification and normalization. In this narrative of stress, it is described as showing up through emotions such as irritability and gloominess, and body sensations such as fatigue and body aches.

#### Stress as “for the weak”

A second theme in FLWs’ narrative of stress is the view that stress only affects those who are “weak.” For FLWs, stress affects only those who are not trained or skilled enough to handle it. For FLWs, being able to handle stress is important not primarily from a mental health perspective, but from a perspective of productivity or the ability to deliver skilled responses (e.g., FLWs fear making costly or devastating mistakes). In this narrative, what is latent is a sense of pride in not letting stress “get to” oneself, and an underlying layer of stigmatization and shame for feeling stressed. FLWs talk about how those who resigned are those who cannot handle the stress, and those who remained are those who can take it. In this narrative, stress is described as behavioral—poor performance, absenteeism, or resignation.

#### Stress as a forbidden cognitive mindset that should not be entertained

A third theme in FLWs’ narrative is a view of stress as damaging, and hence should not be entertained. In their words “Stress will only be ‘stress’ if you think about it as such.” For them, stress is felt only by people who allow themselves to be stressed. Latent in this is a sense of control and responsibility when it comes to emotions. As FLWs, stress is talked about as a dangerous and damaging emotion—with FLWs saying “That’s not allowed for us. We cannot be stressed.” For FLWs, some repercussions of being stressed are self-harm, loss of hope, and suicidality, citing people they know that have gone down this path. Latent in this narrative of stress is its pathologization, and its possible repercussions when it comes to employment, evaluation, and work benefits. In this narrative, stress is understood as a mental health issue and a threat to employability.

### FLWs’ meanings of resilience

The occupational experience, organizational demands, and shared narratives of stress shape contextual meanings of resilience for FLWs.

#### Resilience as ability to endure

FLWs also spoke of resilience as the ability to withstand the pressure of the job and continue to forge on. Given their intertwining narratives of stress as a normal part of their everyday and stress as “for the weak,” FLWs understand resilience as the ability to remain steadfast in the face of daily adversities they face. Here, they likened themselves to a *kalabaw* (a water buffalo), a significant Filipino symbol associated with strength, perseverance, and the ability to withstand back-breaking and strenuous work.

#### Resilience as ability to keep one’s cool

For FLWs, resilience also has an emotional and relational aspect to it. Resilience shows up in being able to keep one’s cool when responding to people in the community, colleagues, and higher-ups. Linked to the narrative of stress as “forbidden cognitive mindset,” FLWs view resilience as the capacity to buffer themselves against getting affected by the stress felt by community members seeking their help, colleagues who have gotten affected by stress, and higher-ups that trigger stress.

#### Resilience as ability to regenerate

FLWs also describe resilience as the “ability to self-heal.” Recognizing that the weight of the work that they deal with day-to-day takes a toll on them, FLWs describe a resilient person as one who can recover and regenerate as they withstand the work. Given their lived experience of work as overwhelming and never-ending, FLWs understand resilience as being able to recover while withstanding ongoing demands.

#### Resilience as ability to separate from work

When regenerating while within work becomes ineffective, FLWs also see that being able to separate from work is resilience as well. FLWs talk about how there are instances where being able to step away from the job (e.g., through taking a leave, setting aside work, or focusing on time with family) helps them regain their ability to handle the workload. In this meaning-making of resilience, FLWs temporarily step away or distance themselves from frontline work that they see as both a “sacrifice and a duty.” While they see frontline work as a responsibility they carry even when not actively on duty, FLWs also recognize the necessity of stepping out of this role, and stepping into more regenerative, supportive, and restful relational spaces (e.g., friends, family, or being with one’s self when engaging with hobbies).

#### Resilience as strength from god’s protection

FLWs also talk about resilience as coming from knowing that they are “shielded by God.” FLWs incorporate aspects of their faith in their day-to-day work, such as passing through churches when they do their rounds in the community, praying, or knowing that their family is praying for them. This spiritual understanding of resilience is both individual and collective. FLWs find strength in their personal religious practices and belief, while also feeling supported by knowing that others keep them in prayer as well.

#### Resilience as collective strength

Most salient in FLWs’ meaning-making is the sense that resilience is something that they draw from each other. In their words “When you are strong, I also feel strong.” FLWs talk about drawing their strength from their colleagues, and from the deep connection that they share, especially as they execute high-risk tasks or demanding responsibilities. FLWs in the field share how they figuratively and literally lean on each other, and how simple gestures like a nod or a glance make them feel ready to face challenges. FLWs’ accounts are also filled with internalized messages of group strength—such as pertaining to their unit as capable, accomplished, or strong (e.g., “Strong 14” as a nickname for their batch of 14 FLWs that were hired during the COVID-19 pandemic and were in charge of setting up the city’s wards and facilities). Hence, for FLWs, part of feeling resilient comes from sensing that their fellow FLWs are also resilient. FLWs also talked about the value of bonding with workmates during and after work, as a way to be well again. In this meaning-making, resilience is understood as relational (shared between fellow FLWs) and embodied (affectively experienced through being with others, sharing a glance, leaning on each other, or a simple nod).

## Discussion

Using a constructivist lens and a systems perspective, this study spotlighted Filipino FLWs’ experiences and meanings of resilience, along with the relationships, spaces, and practices that support or challenge these. This study adds a conceptual contribution to resilience literature in its further exploration of individual and collective resilience. The results of this study are discussed in view of its contributions to the understanding of individual and collective resilience, cultural values and spirituality, and implications for programs and interventions, and institutional support.

### Contextualized view of resilience: individual and collective resilience as intertwined

The findings challenge the dominant framing of resilience that focuses on an individualistic perspective. The current analysis of Filipino FLWs’ experiences of resilience highlights strongly the connection between personal and collective resilience, in that personal resilience is experienced as a process of drawing from the perceived resilience of others or through practices shared with others. In collectivist contexts, such as the Philippines, resilience is seen not only as an individual capacity but as a relational and communal process. This is aligned with the shared cultural values of *kapwa* (shared identity) and *bayanihan* (communal unity), which has *togetherness* as its beginning point (Reyes, 2015). This current analysis is consistent with the conceptualization of collective resilience as a dynamic and relational process rather than a quality of a group, which was demonstrated in how FLWs drew strength from shared struggles and mutual support (Southwick et al., 2014).

Spirituality is found to be another key dimension of resilience as it provides meaning, hope, and strength in adverse circumstances. In this paper, praying for each other or being prayed for by family and loved ones was a salient aspect of FLWs’ resilience practices. The culturally nuanced dimensions underscore collective strength and spirituality, which are often overlooked in mainstream resilience literature. This extends previous work on its role in Filipino well-being (i.e., *kaginhawaan*; Samaco-Zamora and Fernandez, 2016) and aligns with calls for the inclusion of spirituality in psychosocial models of care (Adviento and de Guzman, 2010). Further research could explore how spirituality shapes and supports collective and personal resilience.

### Tensions between narratives of stress and workplace mental health programs

The current study identified tensions between efforts to support workplace mental health vis-a-vis held narratives that normalize, stigmatize, and pathologize stress. The identified narratives of stress highlight blocks in seeking help through the workplace due to perceived or actual repercussions when it comes to employment or career progression. This highlights the importance of understanding deeply embedded beliefs about stress and resilience and considering these in decisions regarding how interventions are to be framed for their intended participants (Oman, 2023). In particular, addressing stigmatizing and pathologizing narratives is key, as well as offering more adaptive constructions of stress.

Furthermore, findings from the current study push resilience interventions to consider how they might support collective or group resilience, as opposed to focusing on supporting individual resilience in a group-based program. Implications of this study point to the value of incorporating group resilience practices, alongside typical individual-focused strategies. While findings have identified the key role that colleagues have in amplifying personal resilience, literature has also identified the important role of leaders in promoting resilience and this could be an important resource to develop as well (Wibowo and Paramita, 2021).

### Critical reflections on resilience

More critically, this study brings to light particular ways of meaning-making of stress and resilience that could lead to normalizing excessive workload, promoting emotional suppression in order to remain efficient at work, minimizing own need for support while continuing to support others, and the under-recognition of institutional responsibility in supporting wellbeing. While personal meaning-making (Sumner and Kinsella, 2021) and social messaging (e.g., calling FLWs as “heroes,” Samadevam, 2022) has been observed to protect against burnout and boost morale, particular narratives about stress and resilience in this study could further the tolerance of difficult circumstances, and place the responsibility on individuals to survive these. These insights align with existing studies that call for reflection on the possible dangers of particular meanings and ways of studying and incorporating resilience in interventions; particularly meanings that are individualized, reductionist, and neoliberal (e.g., Bryant and Aggleton, 2025).

The current findings also present a polyphony of ways of understanding stress, strength, and resilience. Such a multi-layered view of these different meanings move away from simple categorizations of “healthy” or “unhealthy” meanings, by showcasing how different and seemingly contradictory meanings can exist and intertwine within a system. In one narrative, stress can be viewed as “for the weak”—leading to the categorization of people as “weak” or “strong.” In another narrative within the same system, resilience can be viewed as a collective strength—one that can be nourished when people rely on, lean on, and draw strength from one another, breaking dichotomies of “weak” and “strong.”

These findings carry strong implications for interventions. Aligned with previous papers that call to de-individualize the understanding of resilience (e.g., Suslovic and Lett, 2023), current results point to the need for interventions to truly embrace a systems perspective as well – working with leaders and organizations, to push forward the message that resilience is not an individual trait but something that is relational, collective, embodied in shared practices, cultural, and spiritual. Findings offer several entry points for leaders and organizations to reflect on ways to strengthen collective resilience and to reflect on practices and gaps in the system that promote narratives that pathologize stress, discourage help-seeking, and obscure the organization’s responsibility in promoting people’s wellbeing.

### Limitations of the study

This study carries limitations inherent in the decisions made as we constructed the research design. The data collection happened during a single point in time, and may not represent completely the experiences of FLWs at other points in time. The majority of the data involved recollections of past events, which allows for the data to capture meaning-making of those past events. However, these recollections may not fully capture other key aspects or salient thoughts and emotions when those events were unfolding.

The results do not contain an exhaustive representation of the FLWs’ experiences. Rather, it highlights certain aspects of their realities that were co-constructed by the researchers during the research process (Braun and Clarke, 2019, 2023). The researchers are non-FLWs and remain “outsiders” to frontlining work. Although we have engaged in community consultations throughout the research process and employed a reflexive approach in the analysis, being outsiders to the work of FLWs has shaped the data collection and the analysis. Furthermore, ethical considerations regarding data collection and participant recruitment excluded FLWs with low mood and elevated anxiety from participating. Hence, their experience of resilience may be not be captured or represented by the findings of this current study. Future studies can explore meanings and dynamics of resilience particularly among FLWs experiencing greater difficulties and/or vulnerabilities.

As seen in the literature, the meanings of resilience are more contextual rather than universal. While insights gained from this study speak to the experiences of FLWs from a single city in the Philippines, results can be validated with a broader group of FLWs to explore possible resonances with the findings. This study also presented common themes that cut across six different frontline units (police, fire, health, emergency response, social work, and child care and development). Future studies are recommended to explore more deeply the unique ecologies of resilience present in specific frontline units.

## Conclusion

In their day-to-day experience of navigating overwhelming, distressing, and bittersweet moments in their work, Filipino FLWs understand their strength and resilience in many ways—collective, relational, embodied, emotional, cognitive, and spiritual. To further support FLWs, resilience interventions in the workplace need to contend with held narratives that stigmatize and pathologize stress. Results also point to the value of supporting resilience practices of groups and considering spirituality as a resource.

## Data Availability

The datasets presented in this article are not readily available because the participants of this study did not give written consent for their data to be shared publicly, so due to the sensitive nature of the research supporting data is not available. Requests to access the datasets should be directed to Angelique Villasanta, avillasanta@ateneo.edu.
